# Hospital Expenditure—The Commissariat

**Published:** 1897-01-16

**Authors:** 


### The Institutional Workshop.

### HOSPITAL EXPENDITURE—THE COMMISSARIAT.

### XXXIV.—SPECIMEN FORMS.

In forming an accurate estimate of the weekly consumption of food in a hospital, for all practical purposes it is generally sufficient to divide all the supplies under two heads—patients and household. Where but one or two doctors are boarded in the institution, they must be included under the heading of the household. Where an entirely separate table is provided for a large medical staff, the accounts for this branch of expenditure should be kept distinct, and form a third heading.

The returns which should be filled in, in order to gain a thorough grasp of the food consumption under these headings, are neither numerous nor complicated. When once the principle is thoroughly understood, the clerical work is a matter of but a few minutes daily, and in the secretarial department, for which the information thus acquired is destined, all is passed under review, assimilated, and re-issued in the form of weekly, quarterly, and yearly returns. With book-keeping proper, and the accounts defining the cost of the food, we have here no concern. All that relates to the checking of tradesmen's accounts and the entry of expenditure is fully dealt with in "The Uniform System of Accounts," * to which we have on many occasions referred our readers. We are now concerned merely with the system of checking the consumption of food practised in a few hospitals with such conspicuous success, and wholly wanting in many more, mainly, it is believed, for want of a practical knowledge of its working, and the advantages which it offers.

In order to know what food the patients consume it is necessary to know under what diets they are classified. The diets differ from day to day in a manner which to an outsider may appear wholly bewildering. A patient, for instance, admitted under ordinary diet, undergoes an operation which reduces him to milk diet, and subsequently goes through several other gradations before

A. Ward.

Table I.

| | No. of Diets. | | | | | Half-pints. | | | | Extras. | | | | | | | | |
|---|---|---|---|---|---|---|---|---|---|---|---|---|---|---|---|---|---|---|
| | Milk. | Fish. | Ordinary. | Convalescent. | Total. | Milk. | Beef Tea. | Broth. | Ale. | Fowl. | Rabbit. | Eggs. | Jelly. | &c. | „ | | | |
| Sunday ... ... | | | | | | | | | | | | | | | | | | |
| Monday ... ... | | | | | | | | | | | | | | | | | | |
| Tuesday ... ... | | | | | | | | | | | | | | | | | | |
| Wednesday ... | | | | | | | | | | | | | | | | | | |
| Thursday ... | | | | | | | | | | | | | | | | | | |
| Friday ... ... | | | | | | | | | | | | | | | | | | |
| Saturday ... ... | | | | | | | | | | | | | | | | | | |

Sister________________________

Table II.

| | Diets. | | | | | | | | | | | | | | | | | | | Extras. | | | | Alcohol. | | | |
|---|---|---|---|---|---|---|---|---|---|---|---|---|---|---|---|---|---|---|---|---|---|---|---|---|---|---|---|
| | Milk. | Fish. | Ordinary. | Convalescent. | Total. | qts. Milk. | qts. Milk for Puddings. | lbs. Bread. | lbs. Potatoes. | lbs. Butter. | ozs. Tea. | lbs. Sugar. | ozs. Rice. | Diets Fish. | lbs. Meat. | lbs. ditto for Broth. | lbs. ditto, for Beef Tea. | &c. | „ | Fowls. | Rabbits. | Eggs. | &c. | oz. Brandy. | &c. | „ | |
| Ward A ... ... | | | | | | | | | | | | | | | | | | | | | | | | | | | |
| Ward B ... ... | | | | | | | | | | | | | | | | | | | | | | | | | | | |
| Ward C ... ... | | | | | | | | | | | | | | | | | | | | | | | | | | | |
| &c., &c. | | | | | | | | | | | | | | | | | | | | | | | | | | | |
| Totals ... ... | | | | | | | | | | | | | | | | | | | | | | | | | | | |

* "The Uniform System of Accounts, Audit, and Tenders for Hospitals and Institutions," by H. C. Burdett. (Scientific Press, Limited.)

reaching the high-water mark of convalescent diet, and all this in the course of three weeks. Hardly two hospitals agree in the nomenclature or description of their diets; but there are nearly always four or five well-defined varieties, such as milk, low, middle, ordinary, and convalescent. The morning visit of the doctor decides to which diet the patient is assigned for the next twenty-four hours, and the sister of the ward may therefore early in the afternoon get out her requisition of the food required by the patients under her care on the following day. As each diet carries a strictly-defined amount of meat, fish, &c., she will not need to specify the amounts required under each heading, but merely the number of each diet needed. She will enter in detail all extras ordered by the doctor, and append a reckoning of the quantity of milk, beef tea, and broth she will require. Her daily return will be somewhat as indicated in Table I. on the previous page. It must be understood that as all hospitals differ in the degree of variety afforded to the patients we have abbreviated the list as much as possible, knowing that, however lengthy, it could not meet all requirements.

Table III.—Extras.

| | Mr. Z. | | | | | Mr. Y. | | | | | Mr. X., &c. | | | | |
|---|---|---|---|---|---|---|---|---|---|---|---|---|---|---|---|
| | Fowls. | Eggs. | Jelly. | &c. | &c. | Fowls. | Eggs. | Jelly. | &c. | &c. | Fowls. | Eggs. | Jelly. | &c. | &c. |
| Sunday ... ... | | | | | | | | | | | | | | | |
| Monday ... ... | | | | | | | | | | | | | | | |
| Tuesday ... ... | | | | | | | | | | | | | | | |
| Wednesday ... | | | | | | | | | | | | | | | |
| Thursday ... ... | | | | | | | | | | | | | | | |
| Friday ... ... | | | | | | | | | | | | | | | |
| Saturday ... ... | | | | | | | | | | | | | | | |
| Total ... ... | | | | | | | | | | | | | | | |

Signed ____________________ ____________________ ____________________

Table IV. Consumption by Patients.

| Week ending. | Daily Average Number of Patients on Diet. | | | | | Meat. | Fish. | Fowl. | Rabbit. | Eggs. | Milk. | Bread. | Potatoes. | Vegetables. | Butter. | Tea. | Rice, &c. | Malt Liquor. | Wine. | Spirits. |
|---|---|---|---|---|---|---|---|---|---|---|---|---|---|---|---|---|---|---|---|---|
| | Milk. | Fish. | Ordinary. | Convalescent. | Total. | lbs. | lbs. | lbs. | No. | No. | gals | lbs. | lbs. | lbs. | lbs. | lbs. | lbs. | pts. | oz. | oz. |
| Jan. 5th ... ... ... ... | | | | | | | | | | | | | | | | | | | | |
| —— &c. ... ... ... | | | | | | | | | | | | | | | | | | | | |
| —— | | | | | | | | | | | | | | | | | | | | |
| —— | | | | | | | | | | | | | | | | | | | | |
| —— | | | | | | | | | | | | | | | | | | | | |
| &c. | | | | | | | | | | | | | | | | | | | | |
| Total for the Quarter | | | | | | | | | | | | | | | | | | | | |

When these orders are combined they present a daily return of the consumption of food by the patients throughout the hospital; but in the process of putting them together, the weight of meat, fish, &c., is reckoned out from the diets, and entered in detail. Thus, orders to the tradesmen and housekeeper can be prepared from this combined return with absolute accuracy. Table II., on the previous page, is a greatly abbreviated specimen of an excellent form combining the returns from all the wards.

In preparing orders for the tradesmen all that remains to be done is to estimate what weight of butcher's meat must be ordered to provide the meat diets which are reckoned in ounces of cooked meat without bone. This is a mere matter of experience, and presents no difficulty to a practised hand. Fish is commonly ordered from the fishmonger in diets ready prepared of eight ounces. The proportion of beef allowed to each pint of beef-tea and of mutton for broth differs in various hospitals. The amount of meat required for a given number of pints of each is, of course, estimated according to the strength prescribed.

A supplementary return, giving a summary of the extras ordered and indicating by whom the order is given, is found useful in large hospitals. This return is signed weekly by the doctor whose orders it records (see Table III.).

By the mere act of signature the doctor is induced to compare his own estimate of necessary extras with that of his colleagues, and such a mutual comparison can hardly fail to have a good effect in equalising the expenditure for these items and checking extravagance. The materials are now at hand for determining the weekly average consumption of food by the patients. The following return gives a clear summary under the various headings indicated of the quantities of each article consumed for every week in a quarter. Such a summary will be more valuable if the cost of each article is inserted in columns side by side with the amount used (see Table IV.).

Only the roughest outline has been given of the above forms, the design being less to supply model specimens than to illustrate the method of keeping a systematic record of everything used.

